# Schnitzler’s syndrome: lessons from 281 cases

**DOI:** 10.1186/2045-7022-4-41

**Published:** 2014-12-05

**Authors:** Heleen D de Koning

**Affiliations:** Department of Dermatology, Radboud University Medical Center, Nijmegen, The Netherlands; Radboud Institute for Molecular Life Sciences (RIMLS), Nijmegen, The Netherlands; Nijmegen Center for Immunodeficiency and Autoinflammation, Nijmegen, The Netherlands

**Keywords:** Schnitzler’s syndrome, Interleukin-1 beta, Autoinflammation, Chronic urticaria, Paraprotein, Monoclonal gammopathy

## Abstract

Schnitzler’s syndrome is an autoinflammatory disorder characterized by the association of a monoclonal IgM (or IgG) gammopathy, a chronic urticarial rash, and signs and symptoms of systemic inflammation, including fever, arthralgias and bone pain. It was first described in 1972. This review summarizes the clinical features, efficacy of therapies, and follow-up data of the 281 cases that have been reported to date. Also, the results of skin histology, bone imaging, laboratory investigations, and studies of the pathogenesis will be discussed, including the pivotal role of interleukin-1 beta in this disorder.

**Electronic supplementary material:**

The online version of this article (doi:10.1186/2045-7022-4-41) contains supplementary material, which is available to authorized users.

## Introduction

Schnitzler’s syndrome (SchS) is an autoinflammatory disease characterized by the association of a monoclonal immunoglobulin M (IgM, or sometimes IgG) gammopathy (M-protein), a chronic urticarial rash, and signs and symptoms of systemic inflammation [1]. The first case was described by the French dermatologist Professor Dr. Liliane Schnitzler in 1972 [2]. As the phenotype is rather unspecific and many physicians are not familiar with this syndrome, SchS is highly under-diagnosed. This was underlined by the recent retrospective study at the Mayo Clinic, in which 46 undiagnosed cases were identified by cross-referencing cases from their dysproteinemia database with medical records from all patients with chronic urticaria at that institution [3].

This review summarizes the clinical features, efficacy of therapies, and follow-up data regarding the 281 cases that have been reported to date. Also, the results of skin histology, bone imaging, laboratory investigations, and studies on the pathophysiology will be discussed, including the pivotal role of interleukin-1 beta (IL-1β) in this disorder.

## Review

### Methods and diagnostic criteria

In August 2014, a PubMed search using the key words ‘Schnitzler syndrome’ and ‘Schnitzler’s syndrome’ was performed to retrieve all cases. Relevant articles in any language were analyzed, as well as additional cases that are included in the Schnitzler’s syndrome database, which we aim to convert to a registry. In an attempt to prevent double counting of the same patient, the case reports were carefully considered. Cases from the Mayo Clinic, for example, were described in three papers with different focuses. As it could be retrieved from the methods that they mostly overlapped, the cases with IgM monoclonal gammopathy were conservatively regarded as entirely overlapping, and the 4 IgG cases from the Sokumbi paper were added to the 62 IgM patients from the Jain paper [3–5]. Only patients that fulfilled the Strasbourg diagnostic criteria for SchS were included (Table 1). Hence, patients that lacked the paraprotein were excluded, even though they might have developed a monoclonal gammopathy after publication of the case reports.

**Table 1:** **Strasbourg diagnostic criteria for Schnitzler’s syndrome** ^**1**^

**Obligate criteria**	
	Chronic urticarial rash
	Monoclonal IgM or IgG
**Minor criteria**	
	Recurrent fever^2^
	Objective findings of abnormal bone remodeling with or without bone pain^3^
	A neutrophilic dermal infiltrate on skin biopsy^4^
	Leukocytosis and/or elevated CRP^5^
**Definite diagnosis if**	
	Two obligate criteria AND at least two minor criteria if IgM, and three minor criteria if IgG
**Probable diagnosis if**	
	Two obligate criteria AND at least one minor criterion if IgM, and two minor criteria if IgG

^1^Adopted from Simon *et al*., Allergy [1].

^2^A valid criterion if objectively measured. Must be >38°C, and otherwise unexplained. Occurs usually – but not obligatory – together with the skin rash.

^3^As assessed by bone scintigraphy, MRI or elevation of bone alkaline phosphatase.

^4^Corresponds usually to the entity described as ‘neutrophilic urticarial dermatosis’ (Medicine 2009;88:23–31); absence of fibrinoid necrosis and significant dermal edema.

^5^Neutrophils >10 000/mm3 and/or CRP >30 mg/L.

### Epidemiology

**Figure 1:**
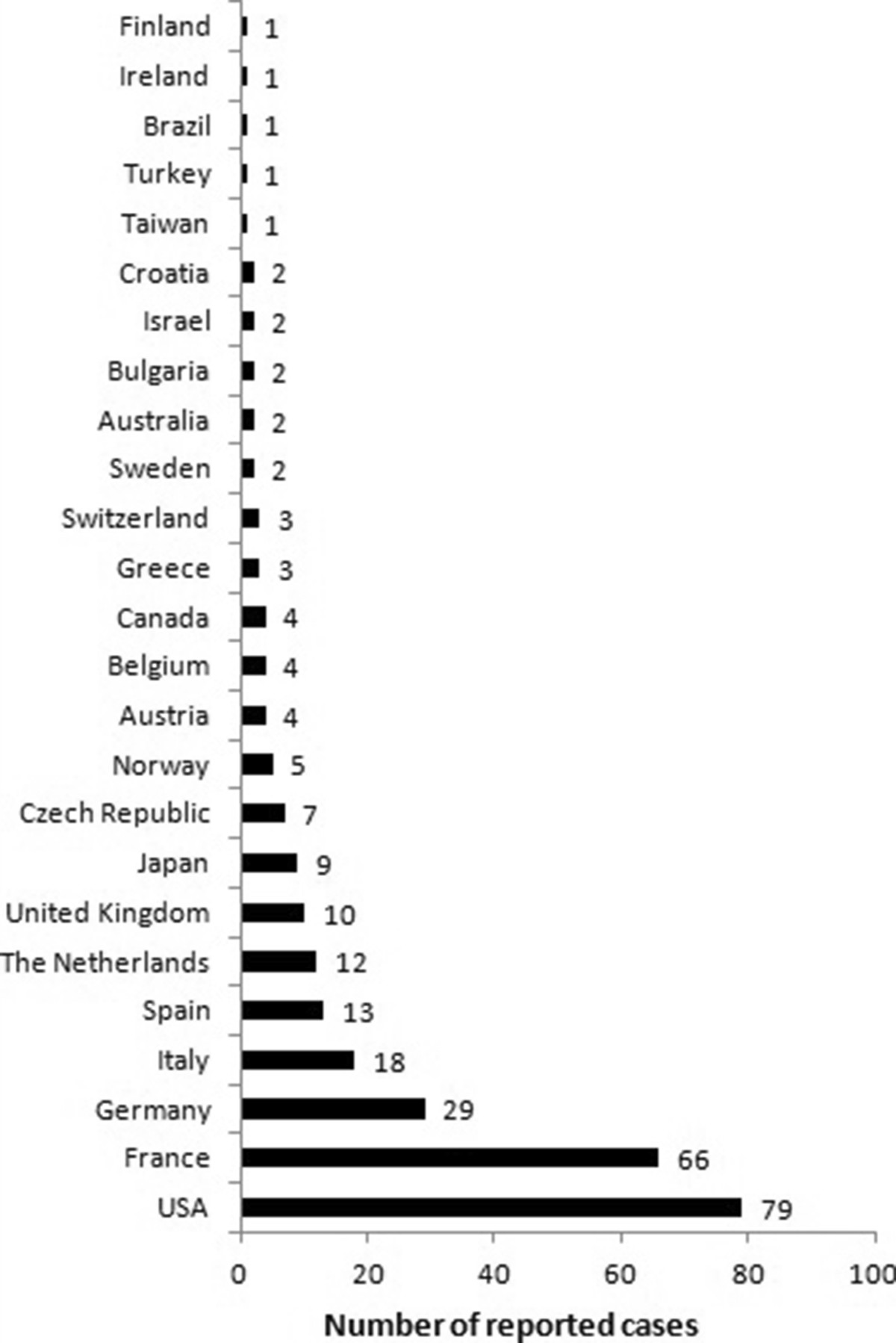
**Number of reported cases per country.**

To date, 281 cases have been reported, with a male–female ratio of 1.5 [2–146]. (Kabashima, Schiff, Frerichs, Van Hoof, Relas, Björk, Fox, Croot: personal communications (p.c.)) As indicated above, SchS is likely to be under-diagnosed, as was illustrated by the study of Jain *et al*. [3] On the other hand, since awareness of this diagnosis is increasing, many cases are not reported unless there are novel findings. Further, as the diagnosis is one of exclusion, one has to carefully consider the differential diagnoses. The median age of onset is 51 years, based on available data on 174 patients. Initially, most cases were reported in French medical journals, but nowadays, patients have been reported in 25 countries including Japan, Australia and Brazil (Figure 1). Most are of Caucasian descent.

### Clinical features

As a major diagnostic criterion, a chronic urticarial rash is present in all patients. In the majority of cases, this is the presenting symptom, and it often precedes the other symptoms several years. Urticaria were explicitly reported as the presenting symptom in 174 cases with a median age of onset of 51 years. The age of onset of the other symptoms was often not mentioned. The age of onset of fever was given in 66 cases, with a median of 52 years old. In 16 of these cases, urticaria preceded the fever (median 3 years; range 1–14 years); in one, fever preceded the urticaria one year; and in the rest, they occurred concurrently. The frequency of urticaria ranges from daily to a few times a year, and the extent of skin involvement differs greatly. Patients describe red skin lesions covering the trunk and extremities, sparing head, neck, palms and soles. Individual lesions persist less than 24 to 48 h. In only 21% of cases, the lesions become pruritic over time, and often the skin lesions are associated with a burning rather than pruritic sensation. Angioedema is rare (Table 2).

**Table 2:** **Clinical features and investigations in Schnitzler’s syndrome**

	Percentage	Number of cases
**Clinical features**		
Chronic urticaria	100%	281
Pruritus	21%	58
Intermittent fever	72%	203
Arthralgia, rarely overt arthritis	68%	192
Bone pain	55%	155
Weight loss	16%	45
Angioedema	8%	22
Lymphadenopathy	26%	72
Hepatomegaly	9%	24
Splenomegaly	6%	17
Neuropathy	7%	20
**Laboratory investigations**		
Elevated ESR/CRP	97%	174/179
Leukocytosis	75%	115/153
Anemia	63%	62/98
***paraprotein***	**100%**	**281**
IgMκ total	85%	232
IgMκ monoclonal	79%	222
IgMλ monoclonal	8%	22
IgGκ monoclonal	5%	13
IgGλ monoclonal	1%	4
IgM, type not specified	2%	7
Ig, type not specified	1%	3
IgMκ & IgMλ	1%	3
IgMκ & IgMκ & IgMλ	0,4%	1
IgMκ & IgGκ	0,4%	1
IgMλ & IgGκ	0,4%	1
IgMκ & IgAκ	0,4%	1
IgMκ & IgAλ	0,4%	1
IgMκ > IgGκ > undetectable	0,4%	1
IgMκ > polyclonal	0,4%	1
κ light chain subtype	89%	249/281
Bence Jones proteins	23%	14/62
**Skin biopsies**		
***dominant histological features***	**100%**	**207**
Neutrophils	50%	104
Lymphocytes +/− macrophages	5%	11
Macrophages	5%	11
Vasculitis	20%	41
Otherwise (mostly unspecified, no vasculitis)	19%	40
***immune deposits***	100%	**83**
None	70%	58
Present	30%	25
IgM	23%	19
IgG	2%	2
C3	14%	12
**Skeletal examinations**		
***conventional radiographs***	100%	**146**
Normal	57%	83
Hyperostosis	39%	57
osteolysis	1%	2
Periostal reaction	0,7%	1
Arthritis	0,7%	1
Abnormal, not specified	1%	2
***scintigraphy***	**100%**	**97**
Normal	13%	13
Increased uptake	85%	82
Infarctions tibiae	1%	1
Osteomyelitis	1%	1
***bone biopsy***	**100%**	**14**
Normal	43%	6
Sclerosis	29%	4
Increased osteoblast & -clast activity	14%	2
Inflammation	7%	1
Aspecific	7%	1
**Bone marrow aspirates or biopsies**	**100%**	**164**
Normal	63%	104
Malignancy*	21%**	34
Plasmacytosis	9%	15
Otherwise (mostly aspecific)	7%	11

Percentages and numbers of patients in which clinical features, laboratory investigations, histological features of skin biopsies, skeletal examinations and bone marrow aspirates were reported.

^*^Specified in Table 4.

^**^Likely to be an overestimation, as in multiple reports, bone marrow aspirates were not mentioned.

The bold numbers indicate the number of cases in which it was reported.

Additional symptoms frequently develop months to years after the disease onset, and often, but not exclusively, occur concurrently with the urticarial rash. The second most common symptom is intermittent fever in 72% of patients, without a periodic pattern. The frequency of fever attacks ranges from daily to a few times a year. Weight loss is reported in 16%. Two-thirds of patients complain of joint pain, mostly in the knees, hips and back, but sometimes also in the hands and feet. Bone pain affects over half of the patients. It typically affects the shins, but also other long bones, the hips and back. Neuropathy was reported in 7% (Table 2). It usually concerns a symmetrical sensory polyneuropathy, and in two patients, anti-myelin-associated-glycoprotein antibodies were found [81, 114]. Single case reports mentioned other symptoms, such as intercostal neuralgia and headache in one patient, and pancreatitis in another, which all ceased during IL-1 blocking treatment [76, 83]. It is unclear if these symptoms belong to the disease spectrum of SchS. In the latter case, this seems unlikely, as the pancreatitis preceded the onset of urticaria of SchS 15 years and the mother and son also suffered from pancreatitis but not from SchS [76]. The response to the IL-1 receptor antagonist (IL-1Ra) anakinra in both cases, suggests involvement of IL-1 in neuropathic pain and pancreatitis, respectively. Other incidentally reported symptoms include pseudoxanthoma elasticum [60, 90, 144], headache, vertigo and depression [60], and severe thrombophilia with antiphospholipid syndrome and hyperhomocysteinemia [51]. The family history is typically negative for the symptoms of SchS.

Physical examination shows a few to numerous nummular erythematous macules or slightly elevated plaques that may become confluent and that cover the trunk and extremities, mostly sparing head and neck, and never involving the palmoplantar skin. These urticarial lesions differ from the wheals of chronic spontaneous and inducible urticarias (CSU and CindU, respectively) in that they are rather symmetrically distributed, persist longer (<24-48 hours per individual lesion), are less edematous, and less itchy [147]. Fever may or may not be present at the time of assessment, and may measure up to 40°C. The affected joints may be painful during examination, but overt arthritis is rarely seen. In only a small number of patients, lymphadenopathy or hepatosplenomegaly are found (Table 2).

### Laboratory investigations

Serum markers of systemic inflammation (erythrocyte sedimentation rate and C-reactive protein) are almost invariably elevated. Leukocytosis, usually neutrophilia, is found in three-quarters of the patients, and anemia in two-thirds (Table 2).

The second hallmark of SchS is a paraprotein, or monoclonal gammopathy, which is of the IgM kappa (IgMκ) subtype in 85% of the patients. Variant cases of the IgG subtype constitute 7% of the reported cases. The actual percentage of IgG cases may be higher, though, as it was initially not included in the definition, and exclusively IgM cases were included in the largest case series [3]. Interestingly, a biclonal gammopathy was present in 7 cases. In 2 of these cases, an IgA monoclonal gammopathy was found in addition to a IgMκ gammopathy. In 1 patient, 3 different IgM M-proteins were found. In 89% of cases, kappa was the light chain subtype. Bence Jones proteins were detected in 23% of the cases in whom they were assessed (Table 2).

As the differential diagnosis of chronic urticaria, a paraprotein, and systemic inflammation is extensive, several hematological, infectious and autoimmune diseases need to be excluded (Table 3). Therefore, laboratory investigations of a patient with these symptoms should include a complete blood count, leukocyte differential, blood cultures, serology for hepatitis C, streptococcal antibodies, rheumatoid factor, antinuclear antibodies, cold agglutinins, cryoglobulins, ferritin, complement factors and serum electrophoresis.

**Table 3:** **Differential diagnosis**

Immunological disorders	
	Adult-onset Still’s disease (AOSD)
	Systemic lupus erythematosus (SLE)
	Acquired C1 esterase deficiency
Hematological disorders	
	Monoclonal gammopathy of unknown significance (MGUS)
	Polyneuropathy, organomegaly, endocrinopathy, monoclonal gammopathy, and skin changes (POEMS) syndrome
	Waldenström’s macroglobulinemia (WM)
	Lymphoma, other than WM
	Multiple myeloma
Hereditary auto-inflammatory syndromes	
	Cryopyrin-associated periodic syndrome (CAPS)
Infectious diseases	
	Hepatitis B, C
	Chronic meningococcemia
Other	
	Chronic spontaneous urticaria
	(Hypocomplementaemic) urticarial vasculitis
	Delayed pressure urticaria
	Cryoglobulinemia
	Erdheim-Chester disease
	Mastocytosis

### Histology of skin biopsies

SchS lesional skin is among the few diagnoses that usually classify as neutrophilic urticarial dermatosis (NUD) [67]. NUD is characterized by a perivascular and interstitial neutrophilic infiltrate with intense leukocytoclasia but without vasculitis and dermal edema. Indeed, in half of the cases, a perivascular and interstitial infiltrate of neutrophils in the absence of vasculitis is reported. Presumably, this is an underestimation, as in 19%, the infiltrate was not specified. Also, sampling errors, treatment effects, or different disease stages could account for the lack of neutrophils in over 10% of cases. Vasculitis was reported in 20% of cases, but this may be an overestimation, as re-evaluation of several of them revealed the absence of fibrinoid necrosis of the vessel walls and thus the lack of vasculitis [148]. Immune depositions consisting of IgM, C3 and sometimes IgG were found in 30% of cases (Table 2). However, the location of the depositions varied greatly, from an interstitial granular pattern to presence along the blood vessel walls (see also “Pathogenesis”).

### Skeletal examinations

Usually, conventional radiographs are taken for the detection of bone abnormalities in SchS. These are normal in over half of the cases, and show osteosclerosis in almost all other cases. Nuclear scintigraphy shows increased uptake in bone in 85% of cases. In most cases, the radiographic and scintigraphic abnormalities are found concomitantly. In others, abnormalities on bone scintigraphy were not found by conventional radiographs, indicating that the former method is more sensitive for detection of the early disease stages [42]. A thorough study in 22 patients with SchS compared the accuracy of several imaging techniques for the detection of bone lesions. Only 38% of osseous abnormalities were identified by plain radiographs, and the remaining ones required the use of other modalities (nuclear scintigraphy, positron emission scanning, gallium scan, magnetic resonance imaging or computed tomography). In view of its high sensitivity, nuclear bone scintigraphy was proposed as the initial screening modality [5]. The most commonly involved sites are the proximal tibia, distal femur, and the innominate bone, but vertebrae, ribs and other bones could also be affected. The focal uptake in the proximal tibiae and distal femora was referred to as the ‘hot knees sign’, which is regarded as typical for both SchS and Erdheim-Chester disease [5]. Bone abnormalities are often found at the site of bone pain. Interestingly, radiographic abnormalities have been reported preceding development of typical clinical symptoms [15]. The few bone biopsies taken were in most cases normal or showed signs of sclerosis (Table 2).

A recent study in serum of 13 SchS patients showed increased bone formation (high bALP, osteocalcin and osteoprotegerin) which was not balanced by an increase in bone resorption (normal CTX and sRANKL). Also, the mean vascular endothelial growth factor (VEGF) levels were almost 3.5-fold higher in SchS compared to controls. Successful treatment led to a significant reduction in VEGF [131].

### Bone marrow examination

Bone marrow aspirates or histology were normal in the majority of cases; plasmacytosis was found in 9%, and a malignancy was found in 21% of the samples. The latter is an overestimation of the actual percentage of malignancies, as in multiple reports, the analyses of bone marrow aspirates were not mentioned. The hematological malignancies are specified in Table 4.

**Table 4:** **Hematological malignancies in patients with Schnitzler’s syndrome**

Malignancies	Cases	Years after onset SchS
Waldenström’s macroglobulinemia	21	1-23, median 8
Lymphoplasmacytic lymphoma, other	3	3,7,?
Non-Hodgkin lymphoplasmocytic mantellar lymphoma	1	?
Non-Hodgkin lymphoma, not specified	2	?
Splenic marginal zone lymphoma	1	8
Marginal zone B-cell lymphoma	1	7
B-cell lymphoma, not specified	2	?,?
Multiple myeloma	2	2,13
Chronic lymphocytic leukemia	1	10
Acute myeloid leukemia	1	?
**Total**	**35**	**median 8 years**

### Differential diagnosis

As SchS is a disorder *per exclusionem*, several autoimmune, hematological, infectious, and other autoinflammatory diseases need to be excluded by means of an extensive workup (Table 3). Depending on the dominant clinical feature, the differential diagnosis can be tailored at either chronic urticaria, recurrent fever, arthralgia/arthritis and/or bone pain, a paraprotein, or a combination. The major considerations are mentioned below.

The most prevalent type of chronic urticaria is CSU, followed by CIU, e.g. cold-induced urticaria. As indicated above, the urticarial lesions in SchS differ from the wheals of these urticarias in that they are rather symmetrically distributed, persist longer (<24-48 hours per individual lesion), are less edematous, less itchy, and rarely associated with angioedema [147]. In contrast to CSU and most CIU, delayed-pressure urticaria can be accompanied by systemic symptoms including fever and arthralgias, but serum markers of inflammation are usually normal. Urticarial vasculitis should be excluded; even though vasculitis was reported in 20% of SchS cases, true fibrinoid necrosis of the vessel walls was absent in 5 re-examined cases, as indicated before. Histologically, NUD is typical for SchS, whereas in CSU and CIU, little leukocytoclasia and much more edema are seen in the dermis [4, 67]. The differential diagnosis of NUD includes other systemic inflammatory diseases, mainly adult-onset Still’s disease (AOSD), lupus erythematosus (LE), and hereditary autoinflammatory syndromes. AOSD is a chronic systemic inflammatory disease characterized by high spiking fever, evanescent rash, pharyngitis, polyarthralgia, lymphadenopathy, hepatosplenomegaly, serositis, and leukocytosis, as well as elevated liver enzymes, erythrocyte sedimentation rate, and serum ferritin associated with low glycosylated fraction of ferritin (<20%) [149]. In AOSD, a pink maculopapular rash occurs rather than the flat urticaria seen in SchS. Bone pain with hyperostosis and a paraprotein are typical for SchS, whereas very high serum ferritin concentrations are rather specific for AOSD. Finally, AOSD primarily affects young adults, in contrast to SchS, in which the median age of onset is 51 years. LE can be accompanied by urticaria, but in systemic LE, the main skin feature is a butterfly-shaped malar erythema, and polycyclic or erythematosquamous plaques in sun-exposed areas in subacute cutaneous LE. Skin biopsies show a lichenoid infiltrate in the majority of cases, but NUD can occur. Arthralgia and fever can occur in LE, but bone pain and a paraprotein are lacking. SchS shares many features with the hereditary autoinflammatory syndrome cryopyrin-associated periodic syndrome (CAPS), which is caused by *NLRP3* mutations that lead to enhanced IL-1β production that induces chronic systemic inflammation, including fever and joint pain. CAPS constitutes a spectrum from cold-induced urticaria to severe neonatal-onset disease with meningitis and debilitating arthritis. In SchS, cold-sensitivity was only mentioned in 3 cases in the literature, but one expert reported this in one-third of their cases. The presence of *NLRP3* variants is not a strong distinguishing factor, as part of the CAPS patients are mutation-negative, and certain *NLRP3* variants were identified in a handful of SchS patients (see “Pathophysiology”). Still, germline NLRP3 mutations are commonly found in CAPS and have not been reported in SchS to date. Depending on the severity, disease onset of CAPS is generally during childhood, and often relatives are affected. Bone pain is usually lacking, and paraproteins have not been reported in CAPS.

The differential diagnosis of the osteosclerotic lesions seen in SchS on conventional radiographs includes Erdheim-Chester disease, metastases, and polyneuropathy, organomegaly, endocrinopathy, monoclonal gammopathy and skin changes (POEMS) syndrome [5]. The so-called ‘hot knees’ sign on bone scintigraphs indicates focal uptake within the distal femora and proximal tibiae, and is very specific for Erdheim-Chester disease, but is often seen in SchS too. The former is a non-Langerhans cell histiocytosis in which several organ systems are affected, leading to neurological signs, bone pain, exophthalmos, and xanthelasmata, among others [150]. POEMS syndrome is characterized by polyradiculoneuropathy, a clonal plasma cell disorder, sclerotic bone lesions, elevated VEGF, the presence of Castleman’s disease, and a few minor criteria. Neuropathy is the dominant feature in POEMS, whereas this was only found in 7% of SchS patients. The paraprotein in POEMS is of the lambda subtype in 91%; in SchS, this is 11% [151]. This suggests notable skewing towards kappa light chain restriction in SchS.

Monoclonal gammopathy of unknown significance (MGUS) is a common disorder and its prevalence increases with age. As chronic urticaria is prevalent as well, one would expect concomitant MGUS in many cases. Kappa and lambda light chains are quite evenly distributed in MGUS, in contrast to the dominance of the kappa light chain in SchS. Jain *et al*. calculated a high odds ratio for a correlation between an IgM monoclonal protein and chronic urticaria (odds ratio 9801; *P* = 0.0001), and stated that chances are high that a patient who has both chronic urticaria and an IgMκ monoclonal gammopathy has SchS [3].

Naturally, the presence of hematopoietic malignancies needs to be excluded by bone marrow examination. Waldenström’s macroglobulinemia (WM) is an important differential diagnosis, but it is also a long-term complication of SchS (see ‘Prognosis’). Thus, even after the diagnosis of SchS is established, one has to remain alert to the development of a lymphoproliferative disorder. The concentration of VEGF cannot help distinguish between SchS, WM, POEMS and IgM MGUS, as it is elevated in all of these disorders [152].

### Treatment

The effects of 35 different treatment modalities have been reported in SchS. Table 5 summarizes the efficacy of all of these, and indicates the number of patients in which it was tried. IL-1 blocking therapies are the most effective ones. Both the IL-Ra anakinra [3, 11, 12, 14, 16, 18, 27, 29, 33, 35, 39–41, 45, 47, 50, 55, 58, 59, 63, 68–71, 76, 77, 83, 92–94, 104, 106, 113, 115, 119–121, 127, 129, 131–133, 135, 136, 140, 153] (Van Hoof, Relas, Björk, Fox, Croot: p.c., and personal observations), which blocks IL-1α and IL-1β, and the anti-IL-1β antibody canakinumab [12, 41, 136, 154] (Fox: p.c.), which only inhibits IL-1β, induce a complete remission in more than 90% of cases. Rilonacept, a fusion protein including the IL-1R, is as effective in 50% of cases [71]. Interestingly, in the 3 cases in which anakinra was not effective, the IL-6 antibody tocilizumab proved highly effective [70]. The anti-CD-20 antibody rituximab, interferon-α, corticosteroids and thalidomide are very effective in about 20% of cases, followed by colchicine and pefloxacine. The latter is partially effective in two-thirds of patients [10, 32, 66, 104, 131]. Corticosteroids were only effective in high doses, the use of which is restricted by the side effects. Thalidomide had to be discontinued in a few cases due to the development of polyneuropathy. The majority of therapies tried were ineffective (Table 5). Antihistamines are invariably ineffective or hardly effective, whereas these drugs are beneficial in histamine-dependent urticarias.

**Table 5:** **Efficacy of therapies tried in Schnitzler’s syndrome**

		Efficacy %		Efficacy, number of cases			Reported
		High	Partial	High	Partial	None	^#^cases
**Highly effective**	anti-IL-1Ra (anakinra)	94%	2%	81	2	3^*^	86
	anti-IL-1β antibodies (canakinumab)	91%	9%	10	1	0	11
	anti-IL-6 antibodies (tocilizumab)	75%	25%	3	1	0	4
	fusion protein IL-1R (rilonacept)	50%	38%	4	3	1	8
**Moderately effective**	anti-CD20 rituximab	21%	16%	4	3	12	19
	IFNα	20%	35%	4	7	9	20
	corticosteroids	18%	46%	33	86	66	185
	thalidomide	19%	25%	3	4	9	16
	colchicine	14%	6%	7	3	41	51
	pefloxacin	13%	63%	2	10	4	16
	cyclosporin	10%	14%	3	4	22	29
**Hardly effective**	PUV-A^1^	8%	62%	1	8	4	13
	alkylating agents	7%	20%	4	12	44	60
	COX inhibitors	6%	33%	6	31	57	94
	hydroxychloroquine	7%	7%	1	1	13	15
	dapsone	5%	5%	2	2	35	39
	histone deacetylase inhibitor (ITF2357)	0%	75%	0	3	1	4
	doxepin	0%	50%	0	3	3	6
	bisphosphonates^2^	0%	33%	0	3	6	9
	I.v. immunoglobulins	0%	25%	0	2	6	8
	psoralen	0%	25%	0	1	3	4
	UVB phototherapy	0%	25%	0	1	3	4
	H1 antihistamine^1^	0%	10%	0	15	132	147
	plasmapheresis	0%	7%	0	1	13	14
	e.c. immunoadsorption	33%	0%	1	0	2	3
	bortezomib	0%	100%	0	1	0	1
	dihydroergotamine	0%	100%	0	1	0	1
**Not effective**	azathioprine	0%	0%	0	0	26	26
	anti-TNF^3^	0%	0%	0	0	10^*^	10
	chloroquine	0%	0%	0	0	6	6
	sulfasalazine	0%	0%	0	0	3	3
	fludarabine	0%	0%	0	0	1	1
	UVA phototherapy	0%	0%	0	0	1	1
	sulphones	0%	0%	0	0	1	1
	leflunomide	0%	0%	0	0	1	1

^1^only against urticaria partially effective.

^2^only against bone pain partially effective.

^3^etanercept, adalimumab, and infliximab were tried.

^*^exacerbation in one case.

The treatment of choice in SchS is anakinra, which is highly effective in 81 of 86 cases. It only suppresses inflammation, and does not cure the disease. Invariably, symptoms recur within 1 to (rarely) 6 days upon withdrawal of anakinra. The major side effect is an erythematous injection site reaction, and upper respiratory tract infections occur more often. As neutropenia may occur during IL-1 inhibition, quarterly leukocyte differential counts are recommended – and shorter intervals during the first weeks after the initiation of treatment. Interestingly, in two SchS cases, IL-1Ra was successfully reintroduced after early occurring treatment-induced neutropenias [104, 136]. As anakinra requires painful daily injections, longer-acting agents are needed. Canakinumab proved to be an effective and safe alternative, but was not registered for SchS [41]. Thus, we need other safe, long-acting IL-1 blocking treatment modalities.

### Follow-up

The major complication of SchS is the development of a hematological malignancy. This was reported in 35 SchS patients at a median follow-up of 8 years after the onset of disease [3, 4, 8, 19, 21, 26, 34, 36, 40, 53, 61, 63, 64, 72, 91, 97, 99, 108, 112, 131, 133, 138, 141]. Two-thirds of these malignancies concerned WM, a lymphoplasmacytic lymphoma (Table 4). Most other cases also developed a lymphoma. Intriguingly, one patient developed acute myeloid leukemia [3]. As exact follow-up data on 74 patients are lacking, the risk of developing a hematological malignancy cannot reliably be indicated. One can only deduce that overall 35 out of 281 reported cases (12%) developed a hematological malignancy. To date, only 1 patient developed WM during IL-1 inhibition: after 2 years of anakinra in combination with methotrexate [63].

Accurate survival data are also lacking, as the duration from disease onset until death is only known for 8 out of the 25 deceased cases, with a median of 6 years. Eighteen patients survived over 20 years after the disease onset. Median follow-up was 7 years. From 58 cases, follow-up data of less than 5 years are available, on top of the 74 cases in which it is unknown. In our previous analysis of the first 94 reported cases, we found no reduction in survival [40]. Jain *et al.* reported that hemoglobin below 12.2 g/dL was the only adverse prognostic factor in their series of 62 patients [3].

One patient developed membranous nephropathy [65]. The development of AA amyloidosis was reported in 6 cases (2%): in one case 5 years after disease onset; in another 10 years, and after an unknown duration in the others [31, 33, 82, 89, 95, 101, 144]. Interestingly, although many SchS patients have been followed up for more than two decades, no cases of AL amyloidosis have been reported.

### Pathogenesis

Theories concerning the pathophysiology of SchS include autoimmunity, a hematological origin, and more recently, autoinflammation. Currently, the latter is the dominant hypothesis.

Initially, most of the cases were reported in dermatological journals, since chronic urticaria is often the presenting symptom of SchS, and professor of Dermatology Liliane Schnitzler was the first to recognize and report the particular combination of chronic urticaria and a monoclonal gammopathy [2]. Consequently, initial investigations focused on the skin by means of histological and immunohistochemical studies. Histopathological examination shows a neutrophilic infiltrate in most cases (Table 1). Hence, a neutrophilic infiltrate on skin biopsy was added as a minor criterium for the diagnosis of SchS [1].

With a monoclonal gammopathy as the second disease hallmark, the presence of immune depositions was assessed in many skin biopsies. It was absent in 70% of the cases, but IgM, C3 and sometimes IgG skin depositions were found in others (Table 1). Still, the location of the depositions varied greatly. No clinical or histological changes were observed upon the injection of purified human IgMκ into rabbit skin [32]. In 2 out of 3 patients, Western blotting of epidermal extracts showed IgMκ anti-skin antibodies which recognized 2 different unknown antigens. This was not seen in dermal abstracts, whereas the immune infiltrate is mostly limited to the dermis [155]. Altogether, the heterogeneous findings render a major causal role for the M-protein in initiating the skin lesions unlikely.

The urticaria are histamine-independent, as antihistamines are invariably ineffective [1, 40], and wheals did not develop upon injection of serum into skin [134]. In one patient, IgG3 antibodies directed against endothelial cells and mast cells, and IgG2 antibodies specific for the alpha-chain of the IgE receptor were detectable, but these antibodies did not mediate histamine release in mast cells or basophils [126].

After the recognition of SchS as a potential autoinflammatory disorder, partly in view of its phenotypical similarities to CAPS, SchS research focused on the role of proinflammatory cytokines, IL-1β in particular [39, 40, 93, 113]. The effect of the IL-1Ra anakinra provided the first evidence for the crucial role IL-1 plays in the pathophysiology of SchS [39, 93]. Further, hypersecretion of IL-1β, IL-6 and TNFα by peripheral blood mononuclear cells (PBMCs) of a patient was found. This could be blocked by the *in-vitro* addition of the caspase-1 inhibitor YVAD, proving involvement of the inflammasome [113]. Others reported a case in which PBMCs sampled during active disease displayed an aberrant spontaneous release of IL-1β that was increased by 2-hour stimulation with LPS. Interestingly, subsequent stimulation of the patient’s cells with the P2X7R agonist BzATP failed to trigger the massive IL-1β release seen in PBMCs from healthy controls. Prednisolone treatment of the patient resulted in diminution of symptoms and normalized the cytokine responses [106]. Similar results were found in the CAPS in which systemic inflammation is caused by activating *NLRP3* mutations. CAPS patient PBMCs constitutively produce IL-1β, and treatment with IL-1Ra results in both a dramatic clinical improvement and substantive down-regulation of LPS-induced IL-1β secretion by the patients’ cells *in vitro*[156]. Such a constitutive IL-1β production was not found by Launay *et al.*, who however did report enhanced LPS-induced IL-1β and IL-6 production by PBMCs of two symptomatic patients, which was reduced by both *in-vitro* and *in-vivo* treatment with anakinra [77]. Migliorini *et al.* found that treatment with IL-1Ra significantly inhibited *IL1B* gene expression in monocytes [94], which indicates the presence of an auto-amplifying loop of IL-1β. Altogether, these data suggest that the inflammasome is primed in monocytes of SchS patients, requiring one rather than the usual two triggers for the activation of IL-1β. Normally, the maturation of IL-1β is tightly controlled, as it is a highly potent proinflammatory cytokine. It induces neutrophilia, anorexia, anemia, fever, chills, skin rash, elevated serum markers of systemic inflammation, joint destruction, and tissue remodeling. IL-1 also induces IL-6 production by e.g. endothelial cells, and IL-6 causes a rise in acute phase reactants, and platelet release from the bone marrow [157]. In 10 SchS cases, elevated serum concentrations of IL-6 were reported [9, 38, 85, 86, 96, 120, 127, 146].

The skin lesions in SchS may well be induced by local IL-1β production in the skin, as Volz *et al.* found IL-1β-expressing dermal cells in one patient [139].

It is not entirely clear how IL-1β overactivation leads to the characteristic bone abnormalities. Both IL-1 and IL-6 stimulate osteoclast function. Possibly, the osteoclerosis seen in SchS results from the elevated VEGF levels, as angiogenesis enhances osteogenesis [131].

The major question regarding the pathophysiology of SchS is if and how the monoclonal gammopathy correlates with the systemic inflammation. Initially, the M-protein was thought to cause the skin symptoms, but as indicated above, this is unlikely. Also, the lack of efficacy of rituximab in most cases shows that lowering the M-protein concentration rarely attenuates the systemic inflammation (Table 5). Further, the calculations of Jain *et al.* show that accidental concomitant occurrence of IgM monoclonal gammopathy and chronic urticaria is not probable [3]. The third option is that the systemic inflammation instigates the formation of a plasma cell clone. This hypothesis is supported by different lines of evidence. First, several cases have been reported in which a M-protein became detectable several years after the onset of symptoms. In a Norwegian patient, for example, this was 13 years [62]. Indeed, a few cases have been reported that lacked the M-protein [137, 158–160]. These were not included in this paper, as only those patients who fulfilled the Strasbourg diagnostic criteria were included, but these may well develop a monoclonal gammopathy over time. Further, in 9 cases, more than one M-protein was present (Table 2), which suggests a common factor capable of inducing plasma cell clones. IL-6 and IL-1β have been implicated in the development of hematological malignancies. IL-6 is a growth factor for B-lymphocytes, and crucial to the growth, proliferation and survival of myeloma cells. Through its stimulation of osteoclast function it also influences the tumor microenvironment in the bone marrow of patients with myeloma [161]. In myeloma, IL-1 was found to stimulate IL-6 release from marrow stromal cells, which stimulates the survival and proliferation of plasma cells. Anakinra decreased IL-6 levels but left numbers of myeloma cells unaffected. However, combination therapy of anakinra with dexamethasone (which induces apoptosis) induced myeloma cell death. Moreover, in some patients with smoldering or indolent multiple myeloma who were at risk of progression to active myeloma, concomitant treatment with IL-1Ra and dexamethasone decreased the myeloma proliferative rate [162]. Thus, speculatively, combination therapy of anakinra with dexamethasone in SchS might clear the malignant clone and M-protein. To date, only one case of reduction of the M-protein concentration during anakinra treatment has been reported in SchS [69]. Neither several years of monotherapy with IL-1Ra, nor several months of treatment with IL-1β antibodies or an IL-1R fusion protein led to a decrease in the monoclonal gammopathy in all other patients [41, 71]. As in general, M-protein levels remain rather stable during anti-IL-1 treatment, one could speculate that IL-1 inhibition is capable of blocking further growth of the plasma cell clone, but cannot induce its demise. The short follow-up during anti-IL-6 treatment did not show a reduction in M-protein concentrations [70]. Long-term follow-up in more patients is needed to determine if IL-6 inhibition can affect the plasma cell clones in SchS. Indeed, anti–IL-6 treatment has been successfully used in Castleman’s disease, a rare lymphoproliferative disorder [163]. Theoretically, chronic infections, such as Epstein Barr virus or cytomegalovirus infections, might play a role in the development of the monoclonal gammopathy, as described in multiple myeloma and WD, but the other symptoms and signs of SchS could not all be accounted for by such an infection. Still, it cannot be excluded as it has not been studied.

In view of the phenotypical similarities to CAPS, and the pivotal role of IL-1β overproduction in both CAPS and SchS, the involvement of the inflammasome in the latter is likely. Analysis of *NLRP3* mutations was negative in 16 of 21 cases [45, 93, 106, 112, 120, 164] (Fox, Kabashima, Relas: p.c.) In two cases, a V198M variant was detected, but both had unaffected family members carrying this variant [88, 112]. It was also reported in families with classical CAPS phenotypes [112], and in patients with autoinflammatory phenotypes who concurrently had mutations in the *Mediterranean fever* gene [165] or a low-penetrance mutation in the *TNFRSF1A* gene [166]. The population allele frequency of this variant is about 0.5%, and as it shows variable expressivity and reduced penetrance, the pathophysiological significance of this variant remains to be determined [112]. In another patient, the Q703K polymorphism was found [127], which is the most common *NLRP3* polymorphism with an allele frequency of 5% in healthy Caucasians [167]. This polymorphism is thus not suspected to play a major inflammation-initiating role, but it could modify inflammation under certain circumstances, as it was reported to lead to gain-of-function alterations [168]. Finally, a clear pathophysiological role of *NLRP3* has just been described in two patients with IgG variant SchS [164]. Our group found in one patient an F523L mutation that was reported to cause a severe neonatal-onset phenotype in two patients with CAPS [169]. The late onset and milder phenotype in the SchS patient is probably due to the restricted occurrence of the *NLRP3* mutation in 10% of the myeloid cells. In our most severely affected SchS patient, we identified a K435E variant in exon 3 of *NLRP3* in about 30% of the myeloid cells. This variant is predicted to be pathogenic, and PBMCs of this patient spontaneously produced high amounts of IL-1β, as did the PBMCs of the patient with the known mutation. Interestingly, the variants were not present in T- or B-lymphocytes, keratinocytes, or fibroblasts of these patients, and presented as myeloid-lineage-restricted mosaicism [164]. Previously, somatic mosaicism of *NLRP3* was reported in neonatal onset CAPS patients [170–173], but there was no significant difference in mutation frequency between several leukocyte subsets and buccal mucosa, which clearly differentiates these patients from the two SchS cases [170]. In a few SchS patients, no mutations were found in the *NLRP1, NOD2*, and *TNFRSF1A* genes [45, 93, 98] (Kabashima, p.c.).

## Discussion

Since our previous review in 2007, the number of reported SchS cases has tripled due to increasing awareness of the disease and the remarkable efficacy of IL-1 inhibition. The focus of research on the pathophysiology of SchS has shifted from hypothetical autoimmune properties of the paraprotein to the autoinflammatory nature of excessive IL-1β production. Further, bone abnormalities have been studied in more detail [5], and an international consensus paper specified the diagnostic criteria, recommended therapies and follow-up [1]. Novel techniques such as NGS provide the opportunity for scrutinizing genetic susceptibility.

We still do not know what triggers the chronic systemic inflammation, but the presence of mosaicism of *NLRP3* mutations in myeloid cells of two variant SchS patients suggests that (mosaicism of) mutations of genes in the IL-1 pathway may be responsible for disease in other cases [164]. NGS will facilitate the detection of even low percentages of mutant cells. The efficacy of anti-IL-6 treatment in 3 patients who were unresponsive to IL-1 inhibition suggests that in some cases, the defect is downstream of IL-1 [70]. This could include aberrant IL-1R signaling or overproduction of IL-6.

The presence of the monoclonal gammopathy is the most puzzling aspect of SchS. Accumulating data suggest that the monoclonal gammopathy is caused by the systemic inflammation rather than vice versa (see ‘Pathophysiology’). In a landmark paper in 2012, Treon *et al.* described a L265P mutation in the *MyD88* gene in bone marrow samples from 49 out of 54 patients with WM. *MyD88* is a crucial adaptor protein for the function of many toll-like receptors and the IL-1R, and the L265P mutation triggered IRAK-mediated NF-κB signaling. In addition, it was associated with a more severe phenotype [174, 175]. As 12% of SchS cases develop WM, the *MyD88* L265P mutation might be present in a subset of SchS patients as well.

Intriguingly, in CAPS, long-term excessive IL-1β signaling does not result in a monoclonal gammopathy. I can only speculate that the high age of onset makes the SchS bone marrow cells more vulnerable for malignant conversion. Hypothetically, the presence of mutant (myeloid) cells in the bone marrow of SchS patients produces high local concentrations of IL-1β and IL-6, facilitating the development of a lymphoproliferative disorder.

During the past 42 years, SchS has evolved from an elusive little-known disorder to an autoinflammatory disorder that is recognized by increasing numbers of dermatologists, rheumatologists, allergologists, hematologists and other specialists. Diagnostic criteria have been revisited, effective treatments have been identified (IL-1 (and IL-6) inhibition), as well as the risk of development of lymphoproliferative disorders, and novel genetic techniques have partially shed light on the pathophysiology of SchS. Presumably, during the next decade, (mosaicism of) mutations of genes in the IL-1 pathway in several other cases of SchS will be uncovered. Finally, long-term follow-up will teach us if IL-1 inhibition is capable of preventing the development of lymphoproliferative disorders.

## Authors’ original submitted files for images

Below are the links to the authors’ original submitted files for images. Authors’ original file for figure 1

## Abbreviations

Ig: Immunoglobulin
IL-1β: Interleukin-1 beta
IL-1Ra: Interleukin-1 receptor antagonist
MGUS: Monoclonal gammopathy of unknown significance
NGS: Next-generation sequencing
NLRP3: Nucleotide-binding oligomerization domain-like receptor family, pyrin domain containing 3
POEMS: Polyneuropathy, organomegaly, endocrinopathy, monoclonal gammopathy and skin changes
SchS: Schnitzler’s syndrome
TNFα: Tumor necrosis factor alpha
VEGF: Vascular endothelial growth factor
WM: Waldenström’s macroglobulinemia.
