# Supplementary figures and images for: Schnitzler’s syndrome: lessons from 281 cases
Source: Clin Transl Allergy. 2014 Dec 5;4:41. doi: 10.1186/2045-7022-4-41 (PMC4405827; doi:10.1186/2045-7022-4-41)

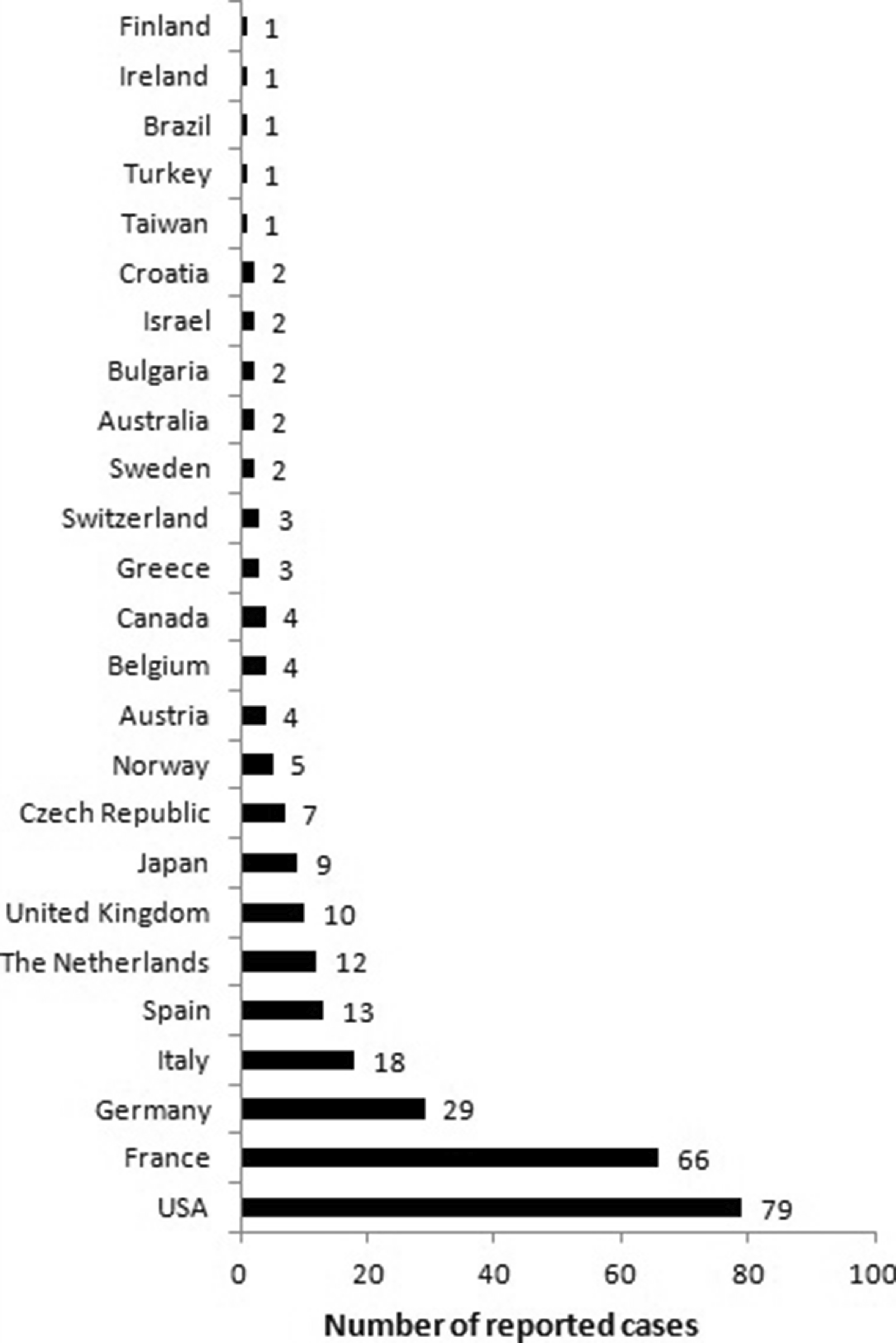

Supplement: Supplementary file 1 — Authors’ original file for figure 1 [file 13601_2014_1073_MOESM1_ESM.tif]
